# Multistate Infestation with the Exotic Disease–Vector Tick *Haemaphysalis longicornis* — United States, August 2017–September 2018

**DOI:** 10.15585/mmwr.mm6747a3

**Published:** 2018-11-30

**Authors:** C. Ben Beard, James Occi, Denise L. Bonilla, Andrea M. Egizi, Dina M. Fonseca, James W. Mertins, Bryon P. Backenson, Waheed I. Bajwa, Alexis M. Barbarin, Matthew A. Bertone, Justin Brown, Neeta P. Connally, Nancy D. Connell, Rebecca J. Eisen, Richard C. Falco, Angela M. James, Rayda K. Krell, Kevin Lahmers, Nicole Lewis, Susan E. Little, Michael Neault, Adalberto A. Pérez de León, Adam R. Randall, Mark G. Ruder, Meriam N. Saleh, Brittany L. Schappach, Betsy A. Schroeder, Leslie L. Seraphin, Morgan Wehtje, Gary P. Wormser, Michael J. Yabsley, William Halperin

**Affiliations:** ^1^Division of Vector-Borne Diseases, National Center for Emerging and Zoonotic Infectious Diseases, CDC; ^2^Center for Vector Biology, School of Environmental and Biological Sciences, Rutgers, The State University of New Jersey, New Brunswick, New Jersey; ^3^Animal and Plant Health Inspection Service, Veterinary Services, U.S. Department of Agriculture, Riverdale, Maryland; ^4^Tick-borne Disease Laboratory, Monmouth County Mosquito Control Division, and Center for Vector Biology, Department of Entomology, Rutgers, The State University of New Jersey, New Brunswick, New Jersey; ^5^Bureau of Communicable Diseases Control, New York State Department of Health; ^6^New York City Department of Health and Mental Hygiene; ^7^Communicable Disease Branch, North Carolina Division of Public Health; ^8^Department of Entomology and Plant Pathology, North Carolina State University, Raleigh, North Carolina; ^9^Pennsylvania Game Commission, Animal Diagnostic Laboratory, Harrisburg, Pennsylvania; ^10^Department of Biological and Environmental Sciences, Western Connecticut State University, Danbury, Connecticut; ^11^Johns Hopkins Center for Health Security, Johns Hopkins Bloomberg School of Public Health, Baltimore, Maryland; ^12^Virginia-Maryland College of Veterinary Medicine, Virginia Polytechnic Institute and State University, Blacksburg, Virginia; ^13^Division of Animal Health, New Jersey Department of Agriculture; ^14^Center for Veterinary Health Sciences, Oklahoma State University, Stillwater, Oklahoma; ^15^Veterinary Division, North Carolina Department of Agriculture and Consumer Services; ^16^Agricultural Research Service, Knipling-Bushland U.S. Livestock Insects Research Laboratory, U.S. Department of Agriculture, Kerrville, Texas; ^17^Animal and Plant Health Inspection Service, Wildlife Services, U.S. Department of Agriculture, Riverdale, Maryland; ^18^Southeastern Cooperative Wildlife Disease Study, Department of Population Health, University of Georgia, Athens, Georgia; ^19^Bureau of Epidemiology, Pennsylvania Department of Health, ^20^Department of Medicine, New York Medical College, Valhalla, New York; ^21^Department of Population Health, College of Veterinary Medicine, and the Warnell School of Forestry and Natural Resources, University of Georgia, Athens, Georgia; ^22^Department of Epidemiology, School of Public Health, Rutgers, The State University of New Jersey, New Brunswick, New Jersey.

*Haemaphysalis longicornis* is a tick indigenous to eastern Asia and an important vector of human and animal disease agents, resulting in such outcomes as human hemorrhagic fever and reduction of production in dairy cattle by 25%. *H. longicornis* was discovered on a sheep in New Jersey in August 2017 (*1*). This was the first detection in the United States outside of quarantine. In the spring of 2018, the tick was again detected at the index site, and later, in other counties in New Jersey, in seven other states in the eastern United States, and in Arkansas. The hosts included six species of domestic animals, six species of wildlife, and humans. To forestall adverse consequences in humans, pets, livestock, and wildlife, several critical actions are indicated, including expanded surveillance to determine the evolving distribution of *H. longicornis*, detection of pathogens that *H. longicornis* currently harbors, determination of the capacity of *H. longicornis* to serve as a vector for a range of potential pathogens, and evaluation of effective agents and methods for the control of *H. longicornis*.

*H. longicornis* is native to eastern China, Japan, the Russian Far East, and Korea. It is an introduced, and now established, exotic species in Australia, New Zealand, and several island nations in the western Pacific Region. Where this tick exists, it is an important vector of human and animal disease agents. In China and Japan, it transmits the severe fever with thrombocytopenia syndrome virus (SFTSV), which causes a human hemorrhagic fever (*2*), and *Rickettsia japonica,* which causes Japanese spotted fever (*3*). Studies in Asia identified ticks infected with various species of *Anaplasma*, *Babesia*, *Borrelia*, *Ehrlichia*, and *Rickettsia*, and all of these pathogen groups circulate zoonotically in the United States (*4*,*5*). In addition, parthenogenetic reproduction, a biologic characteristic of this species, allows a single introduced female tick to generate progeny without mating, thus resulting in massive host infestations. In some regions of New Zealand and Australia, this tick can reduce production in dairy cattle by 25% (*6*). Before 2017, *H. longicornis* ticks were intercepted at U.S. ports of entry at least 15 times on imported animals and materials (James W. Mertins, U.S. Department of Agriculture [USDA], personal communication).

The USDA Animal and Plant Inspection Service coordinated cooperative efforts through telephone conference calls with various local, state, and federal agricultural and public health agencies. Through these efforts, enhanced vector and animal surveillance were implemented to detect additional tick infestations. Suspect archival specimens that were available among previously collected ticks were also examined. Ticks were identified definitively by morphology at the USDA National Veterinary Services Laboratories or by DNA sequence analysis (molecular barcoding) at Rutgers University Center for Vector Biology, Monmouth County (New Jersey) Mosquito Control Division; College of Veterinary Medicine, University of Georgia; and Center for Veterinary Health Sciences, Oklahoma State University. By definition, a “report” is any new morphologic or molecular identification of *H. longicornis* ticks with a new county or host species from that county, identified from August 2017 through September 2018. Subsequent repeat collections are not reported here.

From August 2017 through September 2018, vector and animal surveillance efforts resulted in 53 reports of *H. longicornis* in the United States, including 38 (72%) from animal species (23 [61%] from domestic animals, 13 [34%] from wildlife, and two [5%] from humans), and 15 (28%) from environmental sampling of grass or other vegetation using cloth drags or flags* or carbon dioxide–baited tick traps.^†^ With the exception of one report from Arkansas, the remaining reports of positively identified ticks are from eight eastern states: New Jersey (16; 30%), Virginia (15; 28%), West Virginia (11; 21%), New York (three; 6%), North Carolina (three; 6%), Pennsylvania (two; 4%), Connecticut (one; 2%), and Maryland (one; 2%) (Figure). Among the 546 counties or county equivalents in the nine states, ticks were reported from 45 (8%) counties (1.4% of all 3,109 U.S. counties and county equivalents) (Table 1). Excluding 15 reports of positive environmental sampling using flagging, dragging, or carbon dioxide traps, the remaining 38 reports reflect collection of ticks from infested host species (Table 2). Surveillance efforts did not include testing the ticks or hosts for pathogens. No cases of illness in humans or other species were reported. Concurrent reexamination of archived historical samples showed that invasion occurred years earlier. Most importantly, ticks collected from a deer in West Virginia in 2010 and a dog in New Jersey in 2013 were retrospectively identified as *H. longicornis*.

**FIGURE:**
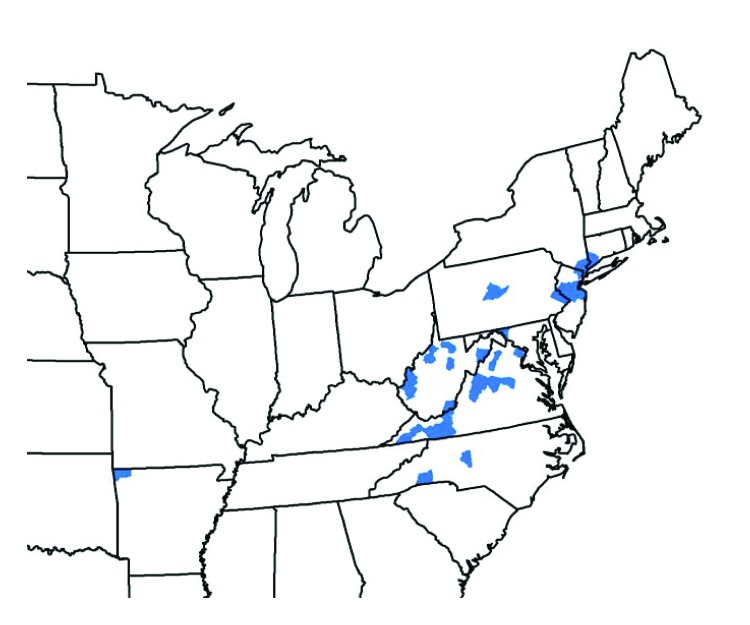
Counties and county equivalents* where *Haemaphysalis longicornis* has been reported (N = 45) — United States, August 2017–September 2018 * Benton County, Arkansas; Fairfield County, Connecticut; Washington County, Maryland; Bergen, Hunterdon, Mercer, Middlesex, Monmouth, Somerset, and Union Counties, New Jersey; Davidson, Polk, and Rutherford Counties, North Carolina; Richmond, Rockland, and Westchester Counties, New York; Bucks and Centre Counties, Pennsylvania; Albemarle, Augusta, Carroll, Fairfax, Giles, Grayson, Louisa, Page, Pulaski, Rockbridge, Russell, Scott, Smyth, Staunton City, Warren, and Wythe Counties, Virginia; Cabell, Hardy, Lincoln, Mason, Marion, Monroe, Putnam, Ritchie, Taylor, Tyler, Upshur Counties, West Virginia.

**TABLE 1:** Percentage of *Haemaphysalis longicornis*–infested counties or county equivalents in infested states — nine states, August 2017–September 2018

State	No. of counties* per state	No. (%) of counties* with *H. longicornis* on host or in environment
Arkansas	75	1 (1)
Connecticut	8	1 (13)
Maryland	24	1 (4)
New Jersey	21	7 (33)
New York	62	3 (5)
North Carolina	100	3 (3)
Pennsylvania	67	2 (3)
Virginia	134	16 (12)
West Virginia	55	11 (20)
**Total**	**546**	**45 (8)**

* Counties or county equivalents

**TABLE 2:** Distribution of *Haemaphysalis longicornis*, by host and species — nine states, August 2017–September 2018

Host category, no. (% of total)/Species	No. (% of host category)
**Domestic animal, 23 (61)**	
Cat	1 (4)
Cow	4 (17)
Dog	12 (52)
Goat	2 (9)
Horse	2 (9)
Sheep	2 (9)
**Total**	**23 (100)**
**Wildlife, 13 (34)**	
Coyote	1 (8)
White-tailed deer	7 (54)
Gray fox	1 (8)
Groundhog	1 (8)
Virginia opossum	2 (15)
Raccoon	1 (8)
**Total**	**13 (100)**
**Human, 2 (5)**	2 (100)
**Total**	**38 (100)**

## Discussion

Cooperative efforts among federal, state, and local experts from agricultural, public health, and academic institutions during the last year have documented that a tick indigenous to Asia is currently resident in several U.S. states. The public health and agricultural impacts of the multistate introduction and subsequent domestic establishment of *H. longicornis* are not known. At present, there is no evidence that *H. longicornis* has transmitted pathogens to humans, domestic animals, or wildlife in the United States. This species, however, is a potential vector of a number of important agents of human and animal diseases in the United States, including *Rickettsia*, *Borrelia*, *Ehrlichia*, *Anaplasma*, *Theileria*, and several important viral agents such as Heartland and Powassan viruses. Consequently, increased tick surveillance is warranted, using standardized animal and environmental sampling methods.

The findings in this report are subject to at least two limitations. First, the findings are limited by the variable surveillance methods used to identify the geographic and host distribution of *H. longicornis.* These methods included both passive and active surveillance. Conclusions about the geographic and host distribution might reflect the biases in the collection and submission of samples to states and USDA and the paucity of available information. Second, the data in this report reflect the collection of specimens that were positively identified by morphology or molecular barcoding. These represent sentinels that *H. longicornis* is present in different U.S. states and regions, and not a comprehensive assessment of the distribution of *H. longicornis* in the United States. The absence of positive samples from many states and counties might reflect the absence of infestation, absence of sampling, or failure to recover the tick. Even in states where *H. longicornis* has been found, the available data do not describe the actual extent or intensity of infestation.

The biology and ecology of *H. longicornis* as an exotic species in the United States should be characterized in terms of its vector competence (ability to transmit a pathogen) and vectorial capacity (feeding habits, host preference, climatic sensitivity, population density, and other factors that can affect the risk for pathogen transmission to humans) for tickborne pathogens known to be present in the United States (*5*). Surveillance for *H. longicornis* should include adequate sampling of companion animals, commercial animals, wildlife, and the environment. Where *H. longicornis* is detected, there should be testing for a range of indigenous and exotic viral, bacterial, and protozoan tickborne pathogens potentially transmitted by *H. longicornis*. Given the similarity between SFTSV and Heartland virus, a tickborne phlebovirus (https://www.cdc.gov/heartland-virus/index.html), further evaluation of the potential role of *H. longicornis* in transmission of this disease agent among animal reservoirs and possibly to humans is warranted. A broad range of interventions should be evaluated, including insecticide and acaricide sensitivity testing. Many state and federal agencies are developing and disseminating information for stakeholders, including development of hotlines, and some states are identifying ticks submitted by the public. The recently documented occurrence of *H. longicornis* in the United States presents an opportunity for collaboration among governmental, agricultural, public health agencies and partners in academic public health, veterinary sciences, and agricultural sciences to prevent diseases of potential national importance before onset in humans and other animal species.

**Summary**

**What is already known about this topic?**

*Haemaphysalis longicornis* is a tick indigenous to Asia, where it is an important vector of human and animal disease agents, which can result in human hemorrhagic fever and substantive reduction in dairy production.

**What is added by this report?**

During 2017–2018, *H. longicornis* has been detected in Arkansas, Connecticut, Maryland, New Jersey, New York, North Carolina, Pennsylvania, Virginia, and West Virginia on various species of domestic animals and wildlife, and from two humans.

**What are the implications for public health practice?**

The presence of *H. longicornis* in the United States represents a new and emerging disease threat. Characterization of the tick’s biology and ecology are needed, and surveillance efforts should include testing for potential indigenous and exotic pathogens.

## Footnotes

* Drags consist of white cloth (usually 1 m^2^) that have a wooden leading frame and are dragged by a cord through grass or a leafy forest floor. Flags are similar but are used to brush uneven surfaces such as small bushes in wooded areas. Drags and flags are used to sample the environment for ticks trying to locate a host.

† Carbon dioxide traps consist of dry ice–filled small boxes with holes that allow the CO_2_ to escape which are placed on a white cloth or mat in a grassy area or forest floor. Ticks, attracted by the CO_2_, crawl on to the cloth or mat surface, which is inspected for ticks after a period of time.

## References

[1] Rainey T, Occi JL, Robbins RG, Egizi A. Discovery of *Haemaphysalis longicornis* (Ixodida: Ixodidae) parasitizing a sheep in New Jersey, United States. J Med Entomol 2018;55:757–9. 10.1093/jme/tjy006 29471482

[2] Luo L-M, Zhao L, Wen H-L, *Haemaphysalis longicornis* ticks as reservoir and vector of severe fever with thrombocytopenia syndrome virus in China. Emerg Infect Dis 2015;21:1770–6. 10.3201/eid2110.150126 26402039 PMC4593435

[3] Mahara F. Japanese spotted fever: report of 31 cases and review of the literature. Emerg Infect Dis 1997;3:105–11. 10.3201/eid0302.970203 9204291 PMC2627607

[4] Kang J-G, Ko S, Smith WB, Kim H-C, Lee I-Y, Chae J-S. Prevalence of *Anaplasma, Bartonella* and *Borrelia* species in *Haemaphysalis longicornis* collected from goats in North Korea. J Vet Sci 2016;17:207–16. 10.4142/jvs.2016.17.2.207 26645342 PMC4921669

[5] Rosenberg R, Lindsey NP, Fischer M, Vital signs: trends in reported vectorborne disease cases—United States and territories, 2004–2016. MMWR Morb Mortal Wkly Rep 2018;67:496–501. 10.15585/mmwr.mm6717e1 29723166 PMC5933869

[6] Heath A. Biology, ecology and distribution of the tick, *Haemaphysalis longicornis* Neumann (Acari: Ixodidae) in New Zealand. N Z Vet J 2016;64:10–20. 10.1080/00480169.2015.1035769 25849758

